# Neutrophil Extracellular Traps (NETs) in Immunity and Diseases: Second Edition

**DOI:** 10.3390/ijms26146563

**Published:** 2025-07-08

**Authors:** Jasmin Knopf

**Affiliations:** 1 Department of Pediatric Surgery, University Medical Center Mannheim, Heidelberg University, 68167 Mannheim, Germany; jasmin.knopf@medma.uni-heidelberg.de; 2 Mannheim Institute for Innate Immunoscience (MI3), Medical Faculty Mannheim, Heidelberg University, 68167 Mannheim, Germany; 3 European Center for Angioscience (ECAS), Medical Faculty Mannheim, Heidelberg University, 68167 Mannheim, Germany

Neutrophils are the most abundant leucocytes in human blood and are the first responders at sites of inflammation or infection. Since the first description of neutrophil extracellular trap formation in 2004 by Brinkmann et al. [1], many advances have been made that have improved our understanding of their contribution to immunity and diseases. As a continuation of the Special Issue “Neutrophil Extracellular Traps (NETs) in Immunity and Diseases”, this Special Issue contains eight research articles focusing on various aspects of NET formation in humans, cattle, canines and mice.

Shukrun et al. [2] compared intestinal biopsies and peripheral blood from pediatric patients with inflammatory bowel disease (IBD) to those from controls and reported significantly higher levels of neutrophils and NETs in IBD patients, especially in those with ulcerative colitis (UC), in both the biopsies and peripheral blood. Interestinlgy, NET formation normalized in treated patients with UC following remission [2]. Czerwińska et al. [3] focused on the involvement of NETs in psoriasis; they measured the levels of neutrophil elastase (NE)-DNA, myeloperoxidase (MPO)-DNA, citrullinated histones (citH2, citH3 and citH4) and DNaseI in the serum of psoriatic patients and compared them at different levels of disease severity. Their results showed that elevated levels of the aforementioned NET parameters correlated with disease severity, and they identified differences in the activity of the NET-degrading enzyme DNaseI. In mild psoriasis, DNaseI was unable to remove NETs due to their suppressing activity, while in moderate to severe psoriasis, the enhanced DNaseI activity was not sufficient to remove NETs upon excessive formation [3]. In the final paper in this subtopic of NET formation in human disease, Vitkov et al. [4] investigated dental biofilm–neutrophil interactions to further elucidate their crucial role in periodontal pathology. They reported that shed acellular dental biofilms consisted mostly of bacterial extracellular vesicles (BEVs), which induced only the moderate formation of NETs in orally healthy subjects, while NET formation was strong in patients with periodontitis. Interestingly, the inhibition of neither interleukin 8 receptor alpha (CXR1) nor interleukin 8 receptor beta (CXR2) had major effects on NET formation. The authors further mechanistically clarified that NET formation by outer membrane vesicles from biofilms is independent of NE, MPO, peptidylarginine deiminase 4 (PAD4) and toll-like receptors (TLR), but rather, occurs via a non-canonical cytosolic LPS/caspase-4/11/Gasdermin D pathway in patients with periodontitis [4].

Maqsood et al. [5] focused on the mechanisms involved in complement-mediated NET formation. They propose that complement-mediated NET formation involves a two-step process: (1) complement deposition followed by neutrophil priming and calcium influx, leading to citH3 formation and attachment to endothelial cells in serum, followed by (2) the NADPH-dependent production of reactive oxygen species (ROS) and NET completion in serum-free conditions [5]. Yasuda et al. [6], on the other hand, focused on the mechanism of NET formation upon induction by the short-chain fatty acid sodium acetate, commonly produced by the gut microbiota and known to enhance immune responses. They investigated histone acetylation by sodium acetate in neutrophil-like HL-60 cells and discovered that the addition of sodium acetate enhanced the acetylation of Ace-H3, H3K9 and H3K14; moreover, this treatment increased NOX-independent NET formation in HL-60 cells but not ROS production. Mechanistically, the addition of the calcium ionophore A23187, a common NET inducer, led to a decrease in histone acetylation, but combined treatment with sodium acetate and A23187 markedly enhanced histone citrullination compared to treatment with A23187 alone, but without increasing the expression of PAD4 [6].

Conejeros et al. [7] investigated the involvement of calcium/calmodulin-dependent protein kinase kinase 2 (CAMKK) and AMP-activated protein kinase (AMPK) in NET formation induced by the intracellular parasite *Besnoitia besnoiti*, the causal agent of bovine besnoitiosis. *B. besnoiti* is known to induce ROS production and NET formation and increase autophagy in bovine neutrophils. The data obtained in their study show the early activation (<30 min) of AMPK in *B. besnoiti*-exposed and AMPK-activator-treated bovine neutrophils, correlating with upstream responses at the level of CAMKK activation. AMPK activator treatment induced both AMPK phosphorylation and NET formation; however, in *B. besnoiti*, the oxidative responses of tachyzoite-exposed neutrophils were not affected, despite enhanced NET formation. The authors conclude that AMPK and autophagy activation synergize in *B. besnoiti*-driven NET formation [7]. The research of León et al. [8] focused on the causes of reproductive failure in dogs. They investigated the interaction between canine neutrophils and spermatozoa and showed, for the first time, that NETs are released in native semen samples and neutrophil/sperm co-cultures. These NETs negatively affect the function of canine spermatozoa by entrapping them, and could therefore represent a cause of reproductive failure in dogs [8]. Yaykasli et al. [9] examined the effect of neutrophil depletion on changes in the glycosylation pattern of immunoglobulin G (IgG) in a murine model of sepsis. Changes in the glycosylation of the IgG Fc region have been reported for several diseases, but the mechanisms remain unclear. The authors report that the glycosylation of the IgG Fc region changed over the course of the disease in the experimental sepsis model and was further influenced by the depletion of neutrophils [9].

This Special Issue contains eight papers that mostly focus on improving our understanding of the contribution of neutrophil and NET formation to the pathology of certain diseases, with three papers also focusing on expanding our knowledge of the mechanisms of NET formation. These studies present exciting new results that open up further avenues for future research. The lack of research articles focusing on understanding how NETs are removed in health and disease emphasizes the need for more research on this aspect of NET formation.

## References

[1] Brinkmann V., Reichard U., Goosmann C., Fauler B., Uhlemann Y., Weiss D.S., Weinrauch Y., Zychlinsky A. (2004). Neutrophil extracellular traps kill bacteria. Science.

[2] Shukrun R., Fidel V., Baron S., Unger N., Ben-Shahar Y., Cohen S., Elhasid R., Yerushalmy-Feler A. (2024). Neutrophil Extracellular Traps in Pediatric Inflammatory Bowel Disease: A Potential Role in Ulcerative Colitis. Int. J. Mol. Sci..

[3] Czerwinska J., Owczarczyk-Saczonek A. (2024). The Impact of Disease Severity on the Serum Levels of Significant Neutrophil Extracellular Trap (NET) Proteins in Patients with Psoriasis. Int. J. Mol. Sci..

[4] Vitkov L., Krunic J., Dudek J., Bobbili M.R., Grillari J., Hausegger B., Mladenovic I., Stojanovic N., Krautgartner W.D., Oberthaler H. (2024). Vesicular Messages from Dental Biofilms for Neutrophils. Int. J. Mol. Sci..

[5] Maqsood M., Suntharalingham S., Khan M., Ortiz-Sandoval C.G., Feitz W.J.C., Palaniyar N., Licht C. (2024). Complement-Mediated Two-Step NETosis: Serum-Induced Complement Activation and Calcium Influx Generate NADPH Oxidase-Dependent NETs in Serum-Free Conditions. Int. J. Mol. Sci..

[6] Yasuda H., Takishita Y., Morita A., Tsutsumi T., Nakagawa N., Sato E.F. (2024). Sodium Acetate Enhances Neutrophil Extracellular Trap Formation via Histone Acetylation Pathway in Neutrophil-like HL-60 Cells. Int. J. Mol. Sci..

[7] Conejeros I., Velasquez Z.D., Rojas-Baron L., Espinosa G., Hermosilla C., Taubert A. (2024). The CAMKK/AMPK Pathway Contributes to *Besnoitia besnoiti*-Induced NETosis in Bovine Polymorphonuclear Neutrophils. Int. J. Mol. Sci..

[8] Leon M., Moya C., Rivera-Concha R., Pezo F., Uribe P., Schulz M., Sanchez R., Taubert A., Hermosilla C., Zambrano F. (2024). Extrusion of Neutrophil Extracellular Traps (NETs) Negatively Impacts Canine Sperm Functions: Implications in Reproductive Failure. Int. J. Mol. Sci..

[9] Yaykasli K.O., van Schie K.A., Toes R.E.M., Wuhrer M., Koeleman C.A.M., Bila G., Negrych N., Schett G., Knopf J., Herrmann M. (2024). Neutrophil Depletion Changes the N-Glycosylation Pattern of IgG in Experimental Murine Sepsis. Int. J. Mol. Sci..

